# A multicenter prospective cohort study to investigate the effectiveness and safety of apixaban in Japanese elderly atrial fibrillation patients (J‐ELD AF Registry)

**DOI:** 10.1002/clc.23294

**Published:** 2019-11-18

**Authors:** Ken Okumura, Takeshi Yamashita, Shinya Suzuki, Masaharu Akao, on behalf of J‐ELD AF Investigators

**Affiliations:** ^1^ Division of Cardiology Saiseikai Kumamoto Hospital Kumamoto Japan; ^2^ Department of Cardiovascular Medicine The Cardiovascular Institute Tokyo Japan; ^3^ Department of Cardiology National Hospital Organization Kyoto Medical Center Kyoto Japan

**Keywords:** anticoagulants, apixaban, atrial fibrillation, elderly, stroke

## Abstract

**Background:**

A global, randomized clinical trial indicated the efficacy and safety of apixaban in stroke prevention in patients with nonvalvular atrial fibrillation (NVAF). However, data in the elderly NVAF patients ≥75 years, especially those on reduced dose, are limited.

**Hypothesis:**

To confirm the current dose reduction criteria of apixaban in elderly NVAF patients.

**Method:**

With a large‐scale, multicenter prospective observational study, one‐year outcomes after administration of on‐label doses of apixaban were analyzed in Japanese NVAF patients aged ≥75 years. Endpoints were stroke or systemic embolism, bleeding requiring hospitalization, total death, and cardiovascular death.

**Results:**

A total of 3031 patients (average age, 81.7 years; female, 48.2%) taking standard (5 mg bid) or reduced dose (2.5 mg bid) of apixaban were enrolled from 110 facilities. Standard and reduced apixaban doses were administered in 1284 (42.4%) and 1747 (57.6%) patients, respectively. Event rates (/100 person‐years) in standard and reduced dose groups were 1.67 and 1.56, respectively, for stroke or systemic embolism, 1.42 and 2.25 for bleeding requiring hospitalization, 1.41 and 4.46 for total death, and 0.41 and 1.36 for cardiovascular death. Reduced apixaban dose was not significantly associated with stroke or systemic embolism and bleeding requiring hospitalization, but was independently associated with total and cardiovascular death.

**Conclusions:**

Incidences of stroke or systemic embolism and bleeding requiring hospitalization were similar between standard and reduced apixaban doses in the elderly NVAF patients. The incidences of total and cardiovascular death were significantly higher in the reduced dose group due to the coexisting higher risks in this group.

## 1 INTRODUCTION

Atrial fibrillation (AF) is one of the major causes of stroke,^1^ and older age is associated with both higher prevalence of AF^2^ and increased incidence of stroke.^3^ Many of the Japanese AF patients are of advanced age (≥75 years)^2^, ^4^, ^5^ and thus at high risk of stroke and systemic embolism.^3^ The same situation is predicted in the world wide in the near future.^6^, ^7^

While warfarin, a vitamin K antagonist, has mainly been used as an oral therapeutic agent in AF for stroke prevention for more than 50 years, its application in elderly patients is not common because of its instability of effect and high incidence rate of serious bleeding.^8^, ^9^ Several direct oral anticoagulants (DOACs) have been developed as alternatives to warfarin^10^ and are currently available worldwide. Apixaban, a direct factor Xa inhibitor, was shown by a worldwide large‐scale randomized clinical trial (ARISTOTLE study) to be effective and safe in stroke prevention for nonvalvular AF patients in comparison with warfarin.^11^

In Asian countries including Japan, AF patients with advanced age often have low body weight and renal dysfunction, being associated with a high risk of bleeding. Thus, many of the elderly AF patients meet the dose reduction criteria configured in each DOAC, although Beers criteria issue a warning on the use of DOACs in these patients.^12^ A recent meta‐analysis of the Asian patients with DOACs in the RE‐LY and ENGAGE AF‐TIMI 48 trials revealed that a risk of stroke or systemic embolism was significantly reduced with standard dose compared with low dose while the rates of major bleeding were similar between the two dosing regimens.^13^ It should be pointed out that in the global ARISTOTLE study, although the benefits of apixaban were consistently observed in patients aged ≥75 years,^14^ a reduced dose (2.5 mg bid) or placebo (ie, warfarin) was administered only to 790 patients (13.9%) among them. Further, in the subanalysis of patients from East Asia (n = 1993),^15^ only 255 patients aged ≥75 years were assigned to apixaban. Thus, evidence of apixaban in the elderly AF patients, especially those on reduced dose, is quite limited. On the other hand, DOAC doses prescribed are often inconsistent with each labeling in real‐world clinical practice, and the off‐label reduced dose of apixaban was shown to be associated with a higher risk of stroke than and no significant difference in major bleeding from the on‐label standard dose.^16^

To elucidate the unmet clinical issue on on‐label doses of apixaban especially on‐label reduced dose in the elderly AF patients, we conducted J‐ELD AF Registry, a large‐scale multicenter prospective observational study on the effectiveness and safety of on‐label doses of apixaban in the Japanese nonvalvular AF patients aged ≥75 years.

## 2 METHODS

### 2.1 Study population

The research physician registered patients who satisfied the selection criteria and did not meet any of the exclusion criteria. The selection criteria were AF patients aged ≥75 years without mitral valve stenosis or prosthetic valve who visited the participating facilities after the start of this study and were taking or started taking apixaban. Exclusion criteria were AF patients with (a) a history of hypersensitivity to apixaban, (b) active bleeding symptoms, (c) liver disease with coagulopathy, (d) creatinine clearance <15 mL/min, and (e) a reduced dose administered although two or more of the dose reduction criteria were not present, which were diagnosed or judged by each investigator. Apixaban was administered according to the package inserts with the reduced dose (2.5 mg bid) when they fulfilled two or more of the following three criteria: ≥80 years of age, body weight <60 kg, and serum creatinine ≥1.5 mg/dL. Otherwise, the patients received the standard dose (5 mg bid). The registration period was from September 2015 to August 2016, and the observation period of each patient was 1 year.

The paucity of previous data on Japanese elderly AF patients taking apixaban made it difficult to set a rational sample size. As this was not a comparative study with warfarin but an exploratory study, to clarify the effectiveness and safety of apixaban in elderly AF patients, the sample size was set to 3000 patients considering the feasibility of the analysis.

### 2.2 Ethics and informed consent

The study design of J‐ELD AF Registry^17^ was published previously (UMIN Clinical Trials Registry: UMIN000017895). The study protocol was approved by the ethics committee of each research facility where the study was conducted. Prior to the registration of patients, the research physician explained the study to the patient using explanatory and agreement documents, and obtained written consent. If the patient withdrew consent during the participation period (observation period) of this study, the study for the patient was discontinued and all existing data collected from the patient were discarded.

### 2.3 Data acquisition

In this study, observation and examination items prescribed in the clinical study implementation plan were collected using an Electronic Data Capture (EDC) system. We collected data on consent acquisition date, age, sex, body weight, underlying diseases (heart failure, hypertension, diabetes, a history of cerebral infarction or transient ischemic attacks [TIAs], myocardial infarction [MI], peripheral artery disease, a history of hospitalization due to bleeding requiring hospitalization, etc.), apixaban dose and its start date, coadministration of antiplatelet drugs, and so on, at the time of registration. The estimated glomerular filtration rate (eGFR) was estimated using the Modification of Diet in Renal Disease study equation: 194 × serum creatinine^‐1.094^ × age^‐0.287^ × 0.739 (if female).^18^ Creatinine clearance was estimated using the Cockcroft‐Gault formulae.^19^

The events were (a) diagnosed stroke with head computed tomography or magnetic resonance imaging with clinical symptoms, (b) systemic embolism confirmed by diagnostic imaging with clinical symptoms, (c) bleeding requiring hospitalization, (d) total death, and (e) cardiovascular death. Patient data were imported into the EDC in an anonymized and unidentifiable state, and were managed by an external third party entrusted by the Cardiovascular Institute Academic Research Organization (CVI ARO).

### 2.4 Evaluation and statistical analysis

The primary endpoints for determining effectiveness and safety were stroke or systemic embolism and bleeding requiring hospitalization, respectively. Secondary endpoints were total death and cardiovascular death. We first calculated the event incidences and their 95% confidence interval (95% CI) by the person‐years method. Moreover, the cumulative event incidences were displayed by the Kaplan‐Meier method, and the differences between groups were tested by the logrank test. Univariate and multivariate models by the Cox regression analysis were then identified. In the multivariate model, apixaban dose was forcibly introduced and then adjusted by the stepwise method with factors composing relevant thromboembolic or bleeding risk scores (ie, CHADS_2_, CHA_2_DS_2_‐VASc, and HAS‐BLED scores): age (≥85 years), male sex, heart failure, hypertension, diabetes mellitus, history of cerebral infarction or TIA, history of MI or peripheral artery disease, history of bleeding requiring hospitalization, liver dysfunction, renal dysfunction (eGFR <45 mL/min/m^2^), habitual drinking, and use of antiplatelet drugs. Among them, the components of age and renal dysfunction were different from original definition of each risk score, but we modified them to secure the statistical power for the adjustment. In all analyses, two‐sided *P* < .05 was taken to indicate statistical significance. Statistical analyses were performed using SAS Ver. 9.4 (SAS Institute Inc., Cary, North Carolina).

## 3 RESULTS

Of 3066 patients registered from 110 nationwide participating institutions (Table S1), 35 were excluded (withdrawal of consent, n = 9; dropout, n = 26), and the remaining 3031 patients (average age, 81.7 years; female, 48.2%) were included in the analyses.

### 3.1 Patient characteristics

Table 1 shows the baseline characteristics of the study population. Of 3031 patients, a standard dose was administered in 1284 (42.4%), while a reduced dose in the remaining 1747 (57.6%). The reduced dose group had a higher age by 5.6 years, higher prevalence of female gender by 1.6 times, lower body weight by 12.1 kg, and lower estimated creatinine clearance by 18.6 mL/min than the standard dose group. The prevalence of pre‐existing heart failure and cerebral infarction or TIAs was also significantly higher in the reduced dose group. The CHADS_2_ scores were 2.8 ± 1.1 and 2.9 ± 1.1 in the standard and reduced dose groups, respectively. Although small, the difference between the two groups was significant (*P* < .001). Similarly, the CHA_2_DS_2_‐VASc scores were 4.2 ± 1.2 and 4.6 ± 1.2 in the standard and reduced dose groups, respectively (*P* < .001). The HAS‐BLED scores were 2.5 ± 0.8 and 2.4 ± 0.8 in the standard and reduced dose groups, respectively (*P* < .001).

**Table 1:** Patient demographics

	Total	Standard dose (5 mg bid)	Reduced dose (2.5 mg bid)	
	(n = 3031)	(n = 1284)	(n = 1747)	*P* value
Gender				<.001
Male, n (%)	1570 (51.8)	855 (66.6)	715 (40.9)	
Female, n (%)	1461 (48.2)	429 (33.4)	1032 (59.1)	
Age, years	81.7 ± 4.6	78.5 ± 3.2	84.1 ± 4.1	<.001
Age ≥ 85 y, n (%)	843 (27.8)	94 (7.3)	749 (42.9)	<.001
Body weight, kg	56.3 ± 11.2	63.3 ± 10.5	51.2 ± 8.6	<.001
Systolic blood pressure, mmHg	127.2 ± 17.4	128.8 ± 16.3	126.1 ± 18.0	<.001
Diastolic blood pressure, mmHg	70.7 ± 12.3	72.8 ± 12.0	69.2 ± 12.3	<.001
Pulse rate, beats/min	74.1 ± 15.0	73.9 ± 15.5	74.3 ± 14.5	.479
Serum creatinine, mg/dL	1.0 ± 0.3	0.9 ± 0.2	1.0 ± 0.4	<.001
Creatinine clearance, mL/min	46.6 ± 16.2	57.3 ± 14.1	38.7 ± 12.8	<.001
Atrial fibrillation types, n (%)				.113
Paroxysmal	1023 (33.8)	407 (31.7)	616 (35.3)	
Persistent	488 (16.1)	225 (17.5)	263 (15.1)	
Permanent	1488 (49.1)	637 (49.6)	851 (48.7)	
Unknown	32 (1.1)	15 (1.2)	17 (1.0)	
EHRA score, n (%)				.144
1	1656 (56.8)	725 (56.5)	931 (53.3)	
2	1059 (36.3)	433 (33.7)	626 (35.8)	
3	174 (6.0)	64 (5.0)	110 (6.3)	
4	27 (0.9)	9 (0.7)	18 (1.0)	
Heart failure, n (%)	1071 (35.3)	338 (26.3)	733 (42.0)	<.001
Hypertension, n (%)	2720 (89.7)	1168 (91.0)	1552 (88.8)	.056
Diabetes mellitus, n (%)	702 (23.2)	336 (26.2)	366 (21.0)	<.001
History of cerebral infarction/TIA n (%)	532 (17.6)	205 (16.0)	327 (18.7)	.049
History of MI/peripheral artery disease, n (%)	287 (9.5)	120 (9.3)	167 (9.6)	.843
History of bleeding requiring hospitalization, n (%)	54 (1.8)	21 (1.6)	33 (1.9)	.678
Liver dysfunction, n (%)	29 (1.0)	13 (1.0)	16 (0.9)	.851
eGFR <45 mL/min/1.73m^2^, n (%)	916 (30.2)	220 (17.1)	696 (39.8)	<.001
Habitual drinking, n (%)	407 (13.4)	259 (20.2)	148 (8.5)	<.001
				
Antiplatelet drugs, n (%)	558 (18.4)	241 (18.8)	317 (18.1)	.670
CHADS_2_ score				
Consecutive	2.8 ± 1.1	2.8 ± 1.1	2.9 ± 1.1	<.001
Category, n (%)				<.001
0	0 (0.0)	0 (0.0)	0 (0.0)	
1	164 (5.4)	72 (5.6)	92 (5.3)	
2	1187 (39.2)	561 (43.7)	626 (35.8)	
3	974 (32.1)	370 (28.8)	604 (34.6)	
4	451 (14.9)	193 (15.0)	258 (14.8)	
5	206 (6.8)	68 (5.3)	138 (7.9)	
6	49 (1.6)	20 (1.6)	29 (1.7)	
CHA_2_DS_2_‐VASc score				
Consecutive	4.4 ± 1.2	4.2 ± 1.2	4.6 ± 1.2	<.001
Category, n (%)				<.001
0	0 (0.0)	0 (0.0)	0 (0.0)	
1	0 (0.0)	0 (0.0)	0 (0.0)	
2	87 (2.9)	48 (5.0)	39 (3.4)	
3	612 (20.3)	359 (37.6)	253 (22.0)	
4	1065 (35.3)	426 (44.7)	639 (55.5)	
5	729 (24.1)	275 (28.8)	454 (39.4)	
6	357 (11.8)	125 (13.1)	232 (20.2)	
7	143 (4.7)	42 (4.4)	101 (8.8)	
8	33 (1.1)	8 (0.8)	25 (2.2)	
9	5 (0.2)	1 (0.1)	4 (0.3)	
HAS‐BLED score				
Consecutive	2.4 ± 0.8	2.5 ± 0.8	2.4 ± 0.8	<.001
Category, n (%)				.005
0	0 (0.0)	0 (0.0)	0 (0.0)	
1	198 (6.6)	73 (7.7)	125 (10.9)	
2	1656 (54.8)	665 (69.7)	991 (86.1)	
3	901 (29.8)	407 (42.7)	494 (42.9)	
4	253 (8.4)	128 (13.4)	125 (10.9)	
5	22 (0.7)	11 (1.2)	11 (1.0)	
6	1 (0.0)	0 (0.0)	1 (0.1)	
7	0 (0.0)	0 (0.0)	0 (0.0)	
8	0 (0.0)	0 (0.0)	0 (0.0)	

Abbreviations: eGFR, estimated glomerular filtration rate; EHRA, European Heart Rhythm Association; MI, myocardial infarction; TIA, transient ischemic attack.

### 3.2 Primary endpoints

#### 3.2.1 Stroke or systemic embolism

**Figure 1:**
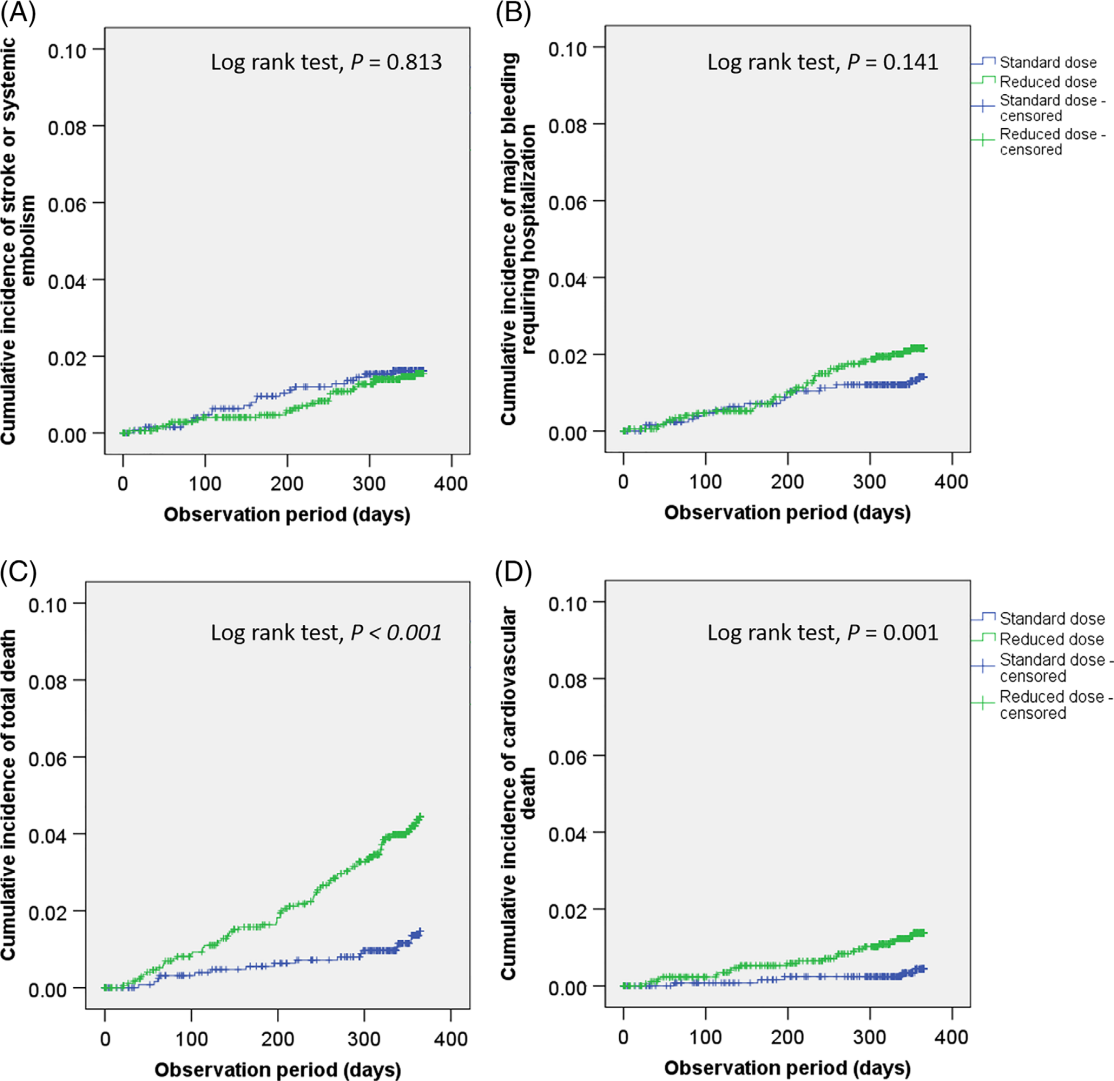
Cumulative incidences shown by Kaplan‐Meier Method. A, Stroke or systemic embolism. B, Bleeding requiring hospitalization. C, Total death. D, Cardiovascular death

The incidence rate of stroke or systemic embolism was 1.67 per 100 person‐years (95% CI: 1.08‐2.58) and 1.56 per 100 person‐years (95% CI: 1.05‐2.30) in the standard and reduced dose group, respectively (logrank test, *P* = .813; Table 2, Figure 1).

**Table 2:** Event incidence rate

	Events	Person‐years	Event rate (95% CI) (/100 person‐years)
Stroke or systemic embolism			
Total	45	2805	1.60 (1.20‐2.15)
Standard dose	20	1197	1.67 (1.08‐2.58)
Reduced dose	25	1608	1.56 (1.05‐2.30)
			
Bleeding requiring hospitalization			
Total	53	2801	1.89 (1.45‐2.47)
Standard dose	17	1198	1.42 (0.89‐2.27)
Reduced dose	36	1602	2.25 (1.62‐3.11)
			
Total death			
Total	89	2821	3.16 (2.56‐3.88)
Standard dose	17	1205	1.41 (0.88‐2.26)
Reduced dose	72	1615	4.46 (3.54‐5.61)
			
Cardiovascular death			
Total	27	2821	0.96 (0.66‐1.39)
Standard dose	*5*	1205	0.41 (0.18‐0.97)
Reduced dose	22	1615	1.36 (0.90‐2.06)

Abbreviations: CI, confidence interval.

In the univariate models of Cox regression analysis, reduced apixaban dose was not significantly associated with stroke or systemic embolism (hazard ratio [HR] 0.93, 95% CI: 0.52‐1.68, *P* = 0.813). In the multivariate model, HR of reduced apixaban dose was 0.91 (95% CI: 0.50‐1.63, *P* = 0.746), and history of cerebral infarction or TIA (HR 2.32, 95% CI: 1.25‐4.32, *P* = 0.008) and history of bleeding requiring hospitalization (HR 4.01, 95% CI: 1.24‐12.94, *P* = 0.020) were independently associated with stroke or systemic embolism (Table 3).

**Table 3:** Cox hazard ratio of the stroke or systemic embolism, and the bleeding requiring hospitalization

	Univariate model		Multivariate model	
	HR (95% CI)	*P* value	HR (95% CI)	*P* value
The stroke or systemic embolism				
Reduced (vs standard) dose	0.93 (0.52‐1.68)	0.813	0.91 (0.50‐1.63)	0.746
Age ≥ 85 years	1.33 (0.71‐2.46)	0.372		
Male sex	1.18 (0.65‐2.13)	0.591		
Heart failure	0.53 (0.26‐1.06)	0.073		
Hypertension	1.63 (0.51‐5.27)	0.412		
Diabetes mellitus	1.07 (0.54‐2.10)	0.855		
History of cerebral infarction/TIA	2.33 (1.26‐4.34)	0.007	2.32 (1.25‐4.32)	0.008
History of MI/PAD	0.95 (0.34‐2.67)	0.930		
History of bleeding requiring Hospitalization	4.11 (1.27‐13.27)	0.018	4.01 (1.24‐12.94)	0.020
Liver dysfunction	1.16 (0.54‐2.49)	0.706		
eGFR <45 mL/min/m^2^	0.85 (0.44‐1.64)	0.623		
Habitual drinking	1.87 (0.92‐3.77)	0.082		
Antiplatelet drugs	1.14 (0.55‐2.36)	0.727		
The bleeding requiring hospitalization				
Reduced (vs standard) dose	1.54 (0.86‐2.75)	0.144	1.33 (0.73‐2.42)	0.348
Age ≥ 85 years	1.53 (0.87‐2.68)	0.142		
Male sex	1.01 (0.59‐1.75)	0.965		
Heart failure	1.06 (0.61‐1.87)	0.827		
Hypertension	0.89 (0.38‐2.08)	0.785		
Diabetes mellitus	0.89 (0.45‐1.72)	0.719		
History of cerebral infarction/TIA	0.97 (0.47‐1.98)	0.926		
History of MI/PAD	2.06 (1.00‐4.22)	0.049		
History of bleeding requiring hospitalization	3.51 (1.09‐11.25)	0.035	3.81 (1.18‐12.23)	0.025
Liver dysfunction	1.44 (0.74‐2.80)	0.283		
eGFR <45 mL/min/m^2^	2.00 (1.16‐3.45)	0.012	1.80 (1.02‐3.17)	0.042
Habitual drinking	1.00 (0.45‐2.23)	0.991		
Antiplatelet drugs	2.02 (1.12‐3.65)	0.019	1.98 (1.09‐3.57)	0.024

Abbreviations: CI, confidence interval; eGFR, estimated glomerular filtration rate; MI, myocardial infarction; PAD, peripheral artery disease; TIA, transient ischemic attack.

#### 3.2.2 Bleeding requiring hospitalization

The incidence of bleeding requiring hospitalization was 1.42 per 100 person‐years (95% CI: 0.89‐2.27) and 2.25 per 100 person‐years (95% CI: 1.62‐3.11) in the standard and reduced dose groups, respectively (logrank test, *P* = 0.141; Table 2 and Figure 1).

In the univariate models of Cox regression analysis for bleeding requiring hospitalization, reduced apixaban dose was not significantly associated with bleeding requiring hospitalization (HR 1.54, 95% CI: 0.86‐2.75, *P* = 0.144). In the multivariate model, HR of reduced apixaban dose was 1.33 (95% CI: 0.73‐2.42, *P* = 0.348), and history of bleeding requiring hospitalization (HR 3.81, 95% CI: 1.18‐12.23, *P* = 0.025), reduced renal function (eGFR <45 mL/min/1.73^2^) (HR 1.80, 95%CI: 1.02‐3.17, *P* = 0.042), and co‐administration of antiplatelet drug (HR 1.98, 95% CI: 1.09‐3.57, *P* = 0.024) were independently associated with bleeding requiring hospitalization (Table 3).

### 3.3 Secondary endpoints

#### 3.3.1 Total death

The incidences of total death were 1.41 per 100 person‐years (95% CI: 0.88‐2.26) and 4.46 per 100 person‐years (95% CI: 3.54‐5.61) in the standard and reduced dose groups, respectively (logrank test, *P* < 0.001; Table 2, Figure 1).

In the univariate models of Cox regression analysis, reduced apixaban dose was significantly associated with total death (HR 3.17, 95% CI: 1.87‐5.38, *P* < 0.001), and the association was independent in the multivariate model (HR 3.19, 95% CI: 1.86‐5.48, *P* < 0.001). Besides the apixaban dose, heart failure (HR 2.48, 95% CI: 1.61‐3.81, *P* < 0.001), male sex (HR 1.92, 95% CI: 1.23‐2.94, *P* = 0.004), and co‐administration of antiplatelet drugs (HR 1.63, 95% CI 1.02‐2.59, *P* = 0.040) were independently associated with total death in the multivariate model (Table S2).

#### 3.3.2 Cardiovascular death

The incidences of cardiovascular death were 0.41 per 100 person‐years (95% CI: 0.18‐0.97) and 1.36 per 100 person‐years (95% CI: 0.90‐2.06) in the standard and reduced dose groups, respectively (logrank test, *P* = .011; Table 2, Figure 1).

In the univariate models of Cox regression analysis, reduced apixaban dose was significantly associated with cardiovascular death (HR 3.30, 95% CI: 1.25‐8.71, *P* < .001), and the association was independent in the multivariate model (HR 3.22, 95% CI: 1.20‐8.67, *P* = .021). Besides the apixaban dose, heart failure (HR 4.65, 95% CI: 1.95‐11.09, *P* = .001), and male sex (HR 2.94, 95% CI: 1.30‐6.67, *P* = .010) were independently associated with cardiovascular death in the multivariate model.

## 4 DISCUSSION

In this study, on‐label doses of apixaban were administered to the Japanese elderly AF patients aged ≥75 years, and one‐year outcomes were prospectively analyzed for standard and reduced dose groups. We found that the incidences of stroke or systemic embolism and bleeding requiring hospitalization after apixaban were both low and similar between the two dose groups. The predictors for stroke or systemic embolism were histories of cerebral infarction/TIAs and bleeding requiring hospitalization, while those for major bleeding were history of bleeding requiring hospitalization, reduced renal dysfunction (eGFR <45 mL/min/m^2^) and coadministration of antiplatelet drug. Importantly, a reduced dose (2.5 mg bid) was not associated with increased risk of either stroke or major bleeding, but was with increased mortality due to higher age and more comorbidities in this group.

### 4.1 Incidences of outcomes in elderly patients under on‐label doses of apixaban

In this J‐ELD AF Registry, we prospectively enrolled and analyzed 3031 patients with an average age of 81.7 years, and included more elderly AF patients than in previous studies.^8^, ^11^, ^20^, ^21^, ^22^, ^23^ The results showed that the event rates of stroke or systemic embolism and bleeding requiring hospitalization were 1.60 and 1.89 per 100 person‐years, respectively. A global ARISTOTLE study showed that the event rate of stroke or systemic embolism and that of major bleeding by International Society on Thrombosis and Haemostasis (ISTH) definition were 1.6%/year and 3.3%/year, respectively, in the patient group assigned to apixaban and with age ≥75 years.^11^ Similar rates for stroke and systemic embolism to and lower rates for bleeding than those in ARISTOTLE study were observed in this study.

In Fushimi AF Registry conducted in Japan, the rates of stroke or systemic embolism and ISTH major bleeding for all patients (mean age, 73.9 years; female, 40.3%; 53.1% anticoagulated) were 2.7 and 1.5 per 100 person‐years, respectively.^20^ Those for patients aged ≥85 years (41.3% anticoagulated) were 5.1 and 2.0 per 100 person‐years, respectively.^3^ In SAKURA‐AF Registry conducted in Japan, 438 patients received apixaban (mean age, 73.2 years; female, 35.8%), and the rates of stroke or systemic embolism and major bleeding were 0.7 and 0.7 at 1 year, and 3.5 and 3.3 at 2 years.^24^ Although the events rates should differ by the patient characteristics of each cohort, our results provide features of Japanese elderly AF patients anticoagulated by on‐label dose of apixaban.

### 4.2 Comparison of outcomes in on‐label standard and reduced doses of apixaban

The patients in the reduced dose group in this study were older, included more females, and had lower body weight and lower estimated creatinine clearance in comparison to the standard dose group. Further, significantly higher rates of previous histories of heart failure and cerebral infarction or TIAs were noted in the reduced dose group. Thus, the reduced dose group in the present study was considered to consist of patients with higher risks of stroke compared with previous studies.^8^, ^11^, ^20^, ^21^, ^22^, ^23^ Meanwhile, the HAS‐BLED score was slightly but significantly lower in the reduced dose group than in the standard dose group.

Despite their estimated higher risk in the reduced dose group, no significant differences in the primary event rates were observed between standard and reduced dose groups. This was consistent with the results of patients aged ≥75 years in the global ARISTOTLE study for standard and reduced dose groups: 1.54%/year and 1.65%/year, respectively, for stroke or systemic embolism, and 3.21%/year and 3.29%/year for ISTH major bleeding, respectively.^14^ On the other hand, this study showed that the reduced dose was significantly associated with higher total death and cardiovascular death. This can be explained by the facts that the reduced dose group had higher age, lower body weight, lower renal function, and higher prevalence of heart failure than the standard dose group, all of which were considered to be associated with higher mortality.

## 5 CLINICAL IMPLICATIONS

The Asian elderly AF patients often have low body weight and low renal function compared with the Western patients,^15^ and therefore, many of them receive a reduced‐dose apixaban. This study revealed the effectiveness and safety of the on‐label reduced dose apixaban in >1700 elderly AF patients, supporting the conclusions shown in the ARISTOTLE subanalysis for the elderly patients.^14^ Since underdosing of apixaban is associated with a higher stroke risk but a similar major bleeding risk,^16^ it would be important to follow the labeling in the elderly AF patients.

## 6 LIMITATIONS

There were several limitations in the present study. First, this J‐ELD AF Registry was a prospective, single‐arm, observational study, and therefore, there was no control arm with which the effect of apixaban was compared. Second, there may have been selection bias for patients. Research physicians might not have enrolled patients at a very high risk who were not suitable for long‐term on‐label doses of apixaban. Third, this study consisted of patients with relatively well‐managed AF since they could be fully followed up by medical institution with cardiovascular specialists on staff. Thus, the patients' health conditions and medical environments may have affected the event incidence rates. Fourth, the observation period was limited to 1 year, and therefore, the results could not be extrapolated to a long‐term clinical course of more than 1 year. Fifth, the outcome events were reported by each participating center, and central adjudication was not performed. However, by simplifying the definition of the stroke and bleeding events, we believe the variation in the local diagnosis between participating centers was modest. Sixth, the status of adherence, discontinuation, or change to other anticoagulants, which would affect the patient outcomes, was not recorded in the present study. Lastly, this study does not include patients taking off‐label underdose, which is common in real‐world clinical practice. The benefit of underdose DOAC is an important matter of debate, but this was not the scope of the present study.

## 7 CONCLUSIONS

We examined the clinical course of the Japanese nonvalvular AF patients with advanced age in whom on‐label doses of apixaban were administered. The incidences of stroke or systemic embolism and bleeding requiring hospitalization were relatively low and were similar between the standard and reduced dose groups. The incidences of total death and cardiovascular death were both significantly higher in the reduced dose group due to the coexisting higher risks in this group.

## CONFLICT OF INTEREST

K.O. received lecture fees from Daiichi‐Sankyo, Boehringer Ingelheim, and Johnson and Johnson. T.Y. received lecture fees from Bristol Myers Squibb, Daiichi‐Sankyo, Bayer, Pfizer, Ono Pharmaceutical, and Toa Eiyo and research funding from Bayer and Daiichi Sankyo. S.S. received research funding from Daiichi‐Sankyo and Mitsubishi‐Tanabe. M.A. received lecture fees from Pfizer, Bristol‐Myers Squibb, Boehringer Ingelheim, Bayer Healthcare, and Daiichi‐Sankyo. This study was conducted by the Cardiovascular Institute Academic Organization (CVI ARO), Tokyo, Japan, subsidized and funded by pharmaceutical and medical device companies. Bristol‐Myers Squibb K.K. provided monetary support for this study. This study was partially supported by the Practical Research Project for Lifestyle‐related Diseases including Cardiovascular Diseases and Diabetes Mellitus from Japan Agency for Medical Research and Development, AMED (15 656 344 and JP17ek0210082). However, there was no conflict of interest between the study center and sponsor concerning the conduct of the study or study outcomes.

## Supporting information




**Table S1** Acknowledgement: 110 Institute participated in J‐ELD AF study

**Table S2** Cox hazard ratio of the total death and the cardiovascular death
