# Supplementary material for: A multicenter prospective cohort study to investigate the effectiveness and safety of apixaban in Japanese elderly atrial fibrillation patients (J‐ELD AF Registry)
Source: Clin Cardiol. 2019 Nov 18;43(3):251–9. doi: 10.1002/clc.23294 (PMC7068106; doi:10.1002/clc.23294)
Supplement: Supplementary file 1 — Table S1 Acknowledgement: 110 Institute participated in J‐ELD AF study [file CLC-43-251-s001.docx]

**Supplementary Table 1. Cox hazard ratio of the total death and the cardiovascular death**

|  | **Univariate model** | | **Multivariate model** | |
| --- | --- | --- | --- | --- |
|  | HR (95% CI) | P value | HR (95% CI) | P value |
| **The total death** | | | | |
| Reduced (vs Standard) dose | 3.17 (1.87–5.38) | <0.001 | 3.19 (1.86–5.48) | <0.001 |
| Age ≥85 years | 2.25 (1.49–3.42) | <0.001 |  |  |
| Male sex | 1.52 (0.99–2.33) | 0.055 | 1.92 (1.23–2.94) | 0.004 |
|  |  |  |  |  |
| Heart failure | 2.85 (1.86–4.35) | <0.001 | 2.48 (1.61–3.81) | <0.001 |
| Hypertension | 1.18 (0.57–2.43) | 0.663 |  |  |
| Diabetes mellitus | 1.15 (0.71–1.85) | 0.569 |  |  |
|  |  |  |  |  |
| History of cerebral  infarction/TIA | 0.65 (0.35–1.22) | 0.182 |  |  |
| History of MI/PAD | 1.97 (1.13–3.43) | 0.017 |  |  |
| History of bleeding requiring  hospitalization | 1.95 (0.62–6.17) | 0.255 |  |  |
|  |  |  |  |  |
| Liver dysfunction | 1.27 (0.75–2.15) | 0.382 |  |  |
| eGFR <45 mL/min/m^2^ | 2.08 (1.37–3.15) | <0.001 |  |  |
| Habitual drinking | 0.64 (0.31–1.32) | 0.229 |  |  |
|  |  |  |  |  |
| Antiplatelet drugs | 1.76 (1.11–2.80) | 0.016 | 1.63 (1.02–2.59) | 0.040 |
|  |  |  |  |  |
|  |  |  |  |  |
| **The cardiovascular death** | | | | |
| Reduced (vs Standard) dose | 3.30 (1.25–8.71) | 0.016 | 3.22 (1.20–8.67) | 0.021 |
| Age ≥85 years | 2.11 (0.99–4.50) | 0.054 |  |  |
| Male sex | 2.22 (0.98–5.00) | 0.057 | 2.94 (1.30–6.67) | 0.010 |
|  |  |  |  |  |
| Heart failure | 5.27 (2.23–12.46) | <0.001 | 4.65 (1.95–11.09) | 0.001 |
| Hypertension | 1.45 (0.34–6.14) | 0.610 |  |  |
| Diabetes mellitus | 1.94 (0.89–4.24) | 0.096 |  |  |
|  |  |  |  |  |
| History of cerebral  infarction/TIA | 0.58 (0.17–1.91) | 0.368 |  |  |
| History of MI/PAD | 2.77 (1.12–6.86) | 0.028 |  |  |
| History of bleeding requiring  hospitalization | 4.48 (1.06–18.90) | 0.041 |  |  |
|  |  |  |  |  |
| Liver dysfunction | 1.54 (0.62–3.80) | 0.354 |  |  |
| eGFR <45 mL/min/m^2^ | 2.50 (1.18–5.32) | 0.017 |  |  |
| Habitual drinking | 0.81 (0.24–2.69) | 0.733 |  |  |
|  |  |  |  |  |
| Antiplatelet drugs | 1.90 (0.83–4.33) | 0.129 |  |  |

TIA, transient ischemic attack; MI, myocardial infarction; PAD, peripheral artery disease; eGFR, estimated glomerular filtration rate; CI, confidence interval.
