# Supplementary material for: A multicenter prospective cohort study to investigate the effectiveness and safety of apixaban in Japanese elderly atrial fibrillation patients (J‐ELD AF Registry)
Source: Clin Cardiol. 2019 Nov 18;43(3):251–9. doi: 10.1002/clc.23294 (PMC7068106; doi:10.1002/clc.23294)
Supplement: Supplementary file 2 — Table S2 Cox hazard ratio of the total death and the cardiovascular death [file CLC-43-251-s002.docx]

**Supplementary Table 2. Acknowledgement: 110 Institute participated in J-ELD AF study**

| Caress Sapporo Hokko Memorial Clinic (Ichiro SAKUMA), Tosei General Hospital (Masayoshi AJIOKA), National Cerebral and Cardiovascular Center (Kengo KUSANO), Tokyo Dental College Ichikawa General Hospital (Takahiro OHKI), Kyoto University Graduate School of Medicine (Takeshi KIMURA), Kyoto Medical Center (Masaharu AKAO), Yamamashi Kousei Hospital (Tetsuya ASAKAWA), Tachikawa General Hospital (Masaaki OKABE), Uwajima City Hospital (Akiyoshi OGIMOTO), Toho University Faculty of Medicine (Takanori IKEDA), Nara Medical University Hospital (Taku NISHIDA), Kameda Medical Center (Akira MIZUKAMI), Tabuchi Cardiovascular Internal Medicine Clinic (Toshifumi TABUCHI), The Cardiovascular Institute (Takeshi YAMASHITA), Honjo Daiichi Hospital (Yasushi SUZUKI ), Kitasato University (Junya AKO), Keio University Hospital (Seiji TAKATSUKI), Kitaishikai Hospital (Takumi SUMIMOTO), Tokyo Women`s Medical University (Nobuhiwa HAGIWARA), Osaka General Medical Center (Takahisa YAMADA), Chikamori Hospital, Chikamori Health Care Group (Masahiko FUKATANI), Kanto Central Hospital (Nobuhiko ITO), Fukuda Memorial Hospital (Kohsuke NAKAJIMA), Japanese Red Cross Society Yamaguchi Hospital (Hiroyuki MICHISHIGE), National Hospital Organization Osaka National Hospital (Yukihiro KORETSUNE), Nakamura Memorial Hospital (Kenji KAMIYAMA), Ogori Daiichi General Hospital (Naoki SUGI), Yamato Municipal Hospital (Takaaki KUBO), Sendai City Hospital (Tetsuo YAGI), Hekinan Municipal Hospital (Atsushi SUGIURA), National Hospital Organization Tokyo Medical Center (Yukihiko MOMIYAMA), Oita Red Cross Hospital (Tetsu IWAO), Yao Municipal Hospital (Tetsuya WATANABE), Yuri Kumiai General Hospital (Tohru NAKANISHI), Fukushima Medical University Hospital (Yasuchika TAKEISHI), Okamisawa Clinic (Kunihiko KAMEDA), Dokkyo Medical University (Teruo INOUE), Saiseikai Kawaguchi General Hospital (Toshikazu FUNAZAKI), Nara Prefectural Seiwa Medical Center (Naofumi DOI), Osaka Police Hospital (Yoshiharu HIGUCHI), Tokyo Metropolitan Geriatric Hospital (Yusuke Tsuboko), Kitakami Saiseikai Hospital (Yoshihiro SATO), Machii Cardiovascular Internal Medicine Clinic (Kazuo MACHII), Tokyo Metropolitan Health and Medical Treatment Corporation Ebara Hospital (Yasunaga HIYOSHI), Ube-kohsan Central Hospital (Masahiko HARADA), Kitano Hospital, Tazuke Kofukai Medical Research Institute (Moriaki INOKO), Hirakata Kohsai Hospital (Hiroyuki TAKENAKA), Kobe City Medical Center General Hospital (Yutaka FURUKAWA), Kokura Memorial Hospital (Kenji ANDO), Tenri Hospital (Yoshihisa NAKAGAWA), Hyogo Prefectural Amagasaki General Medical Center (Kazuyasu YOSHITANI), Shimabara Hospital (Mamoru TAKAHASHI), Mitsubishi Kyoto Hospital (Shinji MIKI), Koto Memorial Hospital (Tomoyuki MURAKAMI), Sakurabashi Watanabe Hospital (Koichi INOUE), Japan Community Health Care Organization Hoshigaoka Medical Center (Yuzuru TAKANO), Nippon Medical School (Wataru SHIMIZU), Daido Hospital (Tomoharu ARAKAWA), Tokushima University Graduate School (Masataka SATA), Mashiko Hospital (Shogo SHIMIZU), Hirosaki Stroke and Rehabilitation Center (Joji HAGII), Kashiwaba Neurosurgical Hospital (Tetsuyuki YOSHIMOTO), Sendai Cardiovascular Center (Shinya Fujii), Faculty of Medicine, Oita University (Naohiko TAKAHASHI), Tsuji Clinic (Hikari TSUJI), Yamato Kashihara Hospital (Tamio NAKAJIMA), Omori Internal Medicine and Cardiology Clinic (Masayuki WATANABE), Fujita Health University School of Medicine (Eiichi WATANABE), Oita Medical Center (Masaya ARIKAWA), Ota Clinic (Akira OTA), Ozaki Cardiology Clinic (Masaharu OZAKI), NHO Kumamoto Saishusou Hospital (Ikuo MISUMI), Kansai Rosai Hospital (Masaaki UEMATSU), Hatayama Clinic (Toru HATAYAMA), SATO Internal Medicine Pediatrics clinic (Atsushi SATO), Narita Medical Clinic (Hidetoshi NARITA), Toyamori Internal Medicine pediatrics clinic (Hiroshi TOYAMORI), St.Mary's Heart Clinic (Kazuhiro YOSHIDA), Furukawa Medical Clinic (Kayoko FURUKAWA), Iwate Medical University (Takashi KOMATSU), Yamaguchi University Graduate School of Medicine (Akihiko SHIMIZU), Shizuoka City Shizuoka Hospital (Tomoya ONODERA), Igarashi Clinic, Cardiology and Internal Medicine (Morio IGARASHI), Fujino Circulation Clinic (Takao FUJINO), Japan Community Health Care Organization Osaka Hospital (Shinji HASEGAWA), Suzuki Internal Medicine Clinic (Tomomi SUZUKI), Odawara municipal hospital (Masaru YUGE), Inoue Cardiology and Internal Medicine Clinic (Takeshi INOUE), Okidate Heart Clinic (Takeshi KUDO), Saien Heart Medical Clinic (Toshihiko KOEDA), Japanese Red Cross Society Fukushima Hospital (Takayuki OHWADA), Sumi Cardiovascular Clinic (Hirokuni SUMI), Takei Clinic (Jinko YAMABE), Iwate Prefectural Kamaishi Hospital (Mahito OZAWA), Medical Corporation Association Koukeikai Sugiura Clinic (Toshiyuki SUGIURA), Hirosaki University Graduate School of Medicine (Ken OKUMURA and Hirofumi TOMITA), Kondo Internal Medicine Clinic (Kazuhiko KONDO), Chiba Surgical Hospital (Yasuhide MORIKAWA), Tokyo Women's Medical University, Yachiyo Medical Center (Shoji HARUTA), ISHIDA Medical Clinic (Shuji ISHIDA), Kindai University Nara Hospital (Manabu SHIROTANI), Nara Prefecture General Medical Center (Fumitaka INOUE), Kumamoto City Hospital (Koji SATO), National Hospital Organization Kumamoto Medical Center (Kazuteru FUJIMOTO), Tohoku Medical and Pharmaceutical University Hospital (Yoshiaki KATAHIRA), Awa Regional Medical Center (Akira MIZUKAMI), Horii Clinic (Norihiko MATSUMURA), JCHO Kumamoto General Hospital (Shinji TAYAMA), Japanese Red Cross Kumamoto Hospital (Ryusuke TSUNODA), Shiga General Hospital Cardiology division (Shigeru IKEGUCHI) |
| --- |
